# High-precision morphology: bifocal 4D-microscopy enables the comparison of detailed cell lineages of two chordate species separated for more than 525 million years

**DOI:** 10.1186/s12915-015-0218-1

**Published:** 2015-12-23

**Authors:** Thomas Stach, Chiara Anselmi

**Affiliations:** Institut für Biologie, Kompetenzzentrum Elektronenmikroskopie, Humboldt-Universität zu Berlin, Philippstrasse 13, Haus 14, 10115 Berlin Germany; Dipartimento di Biologia, Università degli Studi di Padova, Via Ugo Bassi 58/B, 35131 Padova, Italy

**Keywords:** Ascidian, Development, Evolution, Larvacean, Neural crest, Tunicate

## Abstract

**Background:**

Understanding the evolution of divergent developmental trajectories requires detailed comparisons of embryologies at appropriate levels. Cell lineages, the accurate visualization of cleavage patterns, tissue fate restrictions, and morphogenetic movements that occur during the development of individual embryos are currently available for few disparate animal taxa, encumbering evolutionarily meaningful comparisons. Tunicates, considered to be close relatives of vertebrates, are marine invertebrates whose fossil record dates back to 525 million years ago. Life-history strategies across this subphylum are radically different, and include biphasic ascidians with free swimming larvae and a sessile adult stage, and the holoplanktonic larvaceans. Despite considerable progress, notably on the molecular level, the exact extent of evolutionary conservation and innovation during embryology remain obscure.

**Results:**

Here, using the innovative technique of bifocal 4D-microscopy, we demonstrate exactly which characteristics in the cell lineages of the ascidian *Phallusia mammillata* and the larvacean *Oikopleura dioica* were conserved and which were altered during evolution. Our accurate cell lineage trees in combination with detailed three-dimensional representations clearly identify conserved correspondence in relative cell position, cell identity, and fate restriction in several lines from all prospective larval tissues. At the same time, we precisely pinpoint differences observable at all levels of development. These differences comprise fate restrictions, tissue types, complex morphogenetic movement patterns, numerous cases of heterochronous acceleration in the larvacean embryo, and differences in bilateral symmetry.

**Conclusions:**

Our results demonstrate in extraordinary detail the multitude of developmental levels amenable to evolutionary innovation, including subtle changes in the timing of fate restrictions as well as dramatic alterations in complex morphogenetic movements. We anticipate that the precise spatial and temporal cell lineage data will moreover serve as a high-precision guide to devise experimental investigations of other levels, such as molecular interactions between cells or changes in gene expression underlying the documented structural evolutionary changes. Finally, the quantitative amount of digital high-precision morphological data will enable and necessitate software-based similarity assessments as the basis of homology hypotheses.

**Electronic supplementary material:**

The online version of this article (doi:10.1186/s12915-015-0218-1) contains supplementary material, which is available to authorized users.

## Background

The succession of billions of generations of inherited ontogenies with slight modifications from the ontogenies of their predecessors comprises the process of evolution [1, 2]. Comprehension of the evolution of divergent developmental trajectories requires detailed comparisons of embryologies at appropriate levels [3–6]. Cell lineages as detailed and accurate visualizations based on four-dimensional (4D)-microscopy of cleavage patterns, tissue fate restrictions, and morphogenetic movements that occur during the development of individual embryos are currently available for few disparate animal taxa [7–12]. In addition, it is noteworthy that most of the available detailed cell lineages derive from protostomian (sensu [13]) animals encumbering evolutionarily broader comparisons. Tunicates are marine invertebrates considered to be close relatives of vertebrates [14] and whose fossil record dates back to 525 million years ago (Mya) [15, 16]. Life-history strategies across this subphylum are radically different, and include biphasic ascidians with free swimming larvae and a sessile adult stage, the directly developing holoplanktonic larvaceans, and the equally holoplanktonic thaliaceans with some of the most complex life-cycles in the animal kingdom [17]. Despite considerable progress, notably on the molecular level, the exact extent of evolutionary conservation and innovation especially on other organismic levels during embryology remain obscure and leave room for speculation [18, 19]. Here, we overcome the limitations in microscopy imposed by the cellular and acellular coverings of ascidian eggs and embryos by using the innovative technique of bifocal 4D-microscopy. In a comparative approach we demonstrate exactly which characteristics in the cell lineages of the ascidian *Phallusia mammillata* and the previously studied larvacean *Oikopleura dioica* [20] were conserved and which were altered during evolution. Our accurate cell lineage trees combined with the exact three-dimensional reconstructions of cell positions identify clearly the conserved correspondences in cell position, identity, cell movements, and fate restrictions in several cell lines while at the same time precisely pinpointing differences observable at all levels of development. These differences comprise fate restrictions, tissue types, complex morphogenetic movement patterns, bilateral asymmetry, and numerous cases of heterochronous acceleration in the larvacean embryo. Our results demonstrate in extraordinary detail the multitude of developmental levels amenable to evolutionary innovation. We anticipate that the detailed cell lineage data combined with the accurate relative spatial representation of cells will moreover serve as a high-precision guide to devise experimental investigation of other levels, such as molecular interactions between cells or changes in gene expression underlying the documented structural evolutionary changes of ontogenetic processes. Finally, the sheer amount of digital high-precision morphological data will enable and necessitate new attempts to formulate software-based, quantifiable similarity assessments as the basis of homology hypotheses.

## Results and discussion

**Fig. 1:**
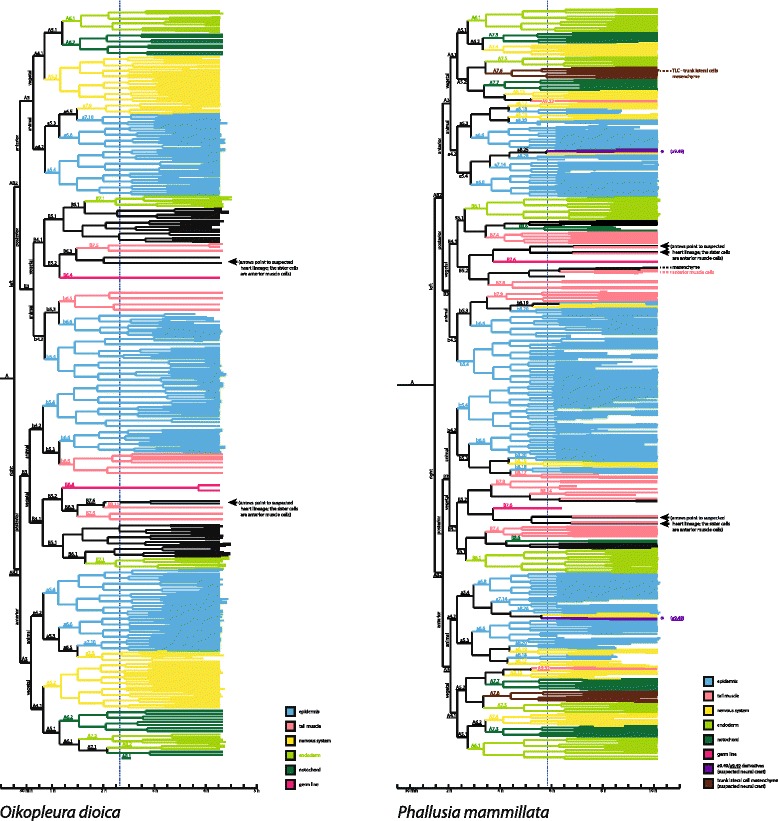
Cell lineage trees of the developments of a larvacean (*left*, *Oikopleura dioica*) and an ascidian (*right*, *Phallusia mammillata*) embryo. Different colors denote different tissue fates. Note the different time scales. *Blue dotted line* indicates respective timeline of stages depicted in Fig. 4. For the *black lines*, tissue fate was either not restricted to a single fate yet or a confident determination was not possible at the latest stage documented. Branches in the trees are arranged according to the same scheme (see first paragraph of “Results and Discussion” for details)

Bifocal 4D-microscopy considerably extends the range of focus compared to conventional 4D-microscopy [11] and is therefore useful in larger embryos, more opaque embryos, or in embryos that are invested with protective coverings. At least the latter, and usually a combination of these limiting factors, are present in ascidian embryos. Ascidian eggs and embryos are protected during their development by an outer layer of follicle cells, a chorion membrane, and an inner layer of chorion cells [21, 22]. The simultaneous comparative analysis of two tunicate species using the same analytical software (Simi Biocell, Simi Reality Motion Systems GmbH, Unterschleißheim, Germany) and a similar recording technique, and therefore the same level of precision, allowed for an improved re-analysis of the previously reported cell lineage of the larvacean *Oikopleura dioica* (Fig. 1a), enabling improvements over the original cell lineage results reported in [20] for the larvacean, while adding a cell lineage in an ascidian species, *Phallusia mammillata* (Fig. 1b), at unprecedented precision. Based on this comparative approach, it was possible to identify the progeny of A6.1 in *O. dioica* as purely endodermal and descendants of B8.12/B8.12 as probable heart precursor cells. Because no mesenchymal cells, such as blood cells or tunic cells, are present in the adult larvacean and because the heart, surrounded by purely epithelial cells, is the only mesodermal structure in the adult larvacean [23, 24], we suspect that all descendants of B5.1/B5.1 are purely endodermal in their respective fates. However, because we could not rule out the presence of mesenchymal cells in the 4 h 30 min old hatchling on the basis of the present light-microscopic investigation, we cautiously refrained from denoting all B5.1/B5.1 descendants as endodermally fate restricted (Fig. 1a).

In order to facilitate comparisons between trees, we used Conklin’s nomenclature for both species [20, 25]. Conklin also devised a set of rules that assures comparability that we followed in our analyses and applied to the tree [25, 26] depictions (for more information, see “Methods”). To distinguish between the cells from both sides, we again used Conklin’s nomenclature by underlining cell names referring to the right side of the embryo.

While the ascidian cell lineage tree is highly symmetrical (Fig. 1b), the larvacean cell lineage tree shows a marked asymmetry in the lineage of the notochord. On the left side, A8.1 as a descendant of A6.1 is already restricted to an endodermal fate. On the right side, A8.1 gives rise to additional notochordal cells.

The direct side-by-side comparison of the 4D-microscopical cell lineage trees of the ascidian *P. mammillata* and the larvacean *O. dioica* (Fig. 1) reveals exact correspondences and differences of various degrees. In the following, we discuss examples of these different relations. It should be noted, that more examples are documented in the extensive supplementary material accompanying this publication online.

### Exact correspondences of cell identity and fate

**Fig. 2:**
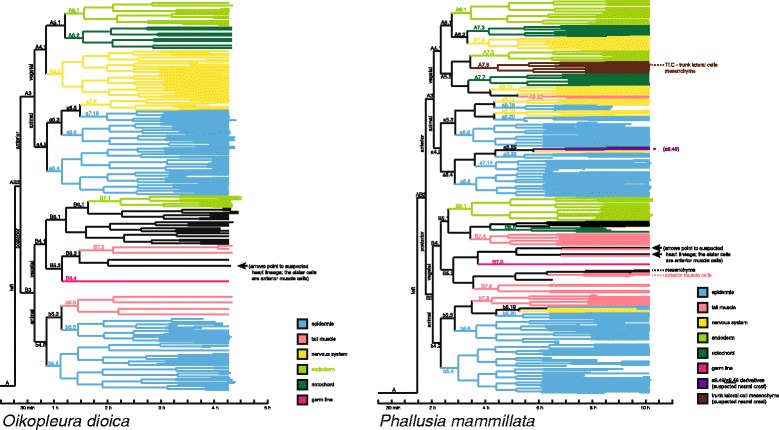
To increase resolution, only the upper halves (left of the embryo) of cell lineage trees from Fig. 1 are magnified. *Left*: the larvacean *Oikopleura dioica*, *right*: the ascidian *Phallusia mammillata*. Different colors denote different tissue fates. Note the different time scales. For the *black lines*, tissue fate was either not restricted to a single fate yet or a confident determination was not possible at the latest stage documented. Branches in the trees are arranged according to the same scheme (see first paragraph of “Results and Discussion” for details)

Exact correspondences in relative position, cell identity, and fate in both species are observed in several cells. Cells A6.1/A6.1 and B7.1/B7.1 are already restricted to become the progenitors of endodermal cellular fates by the sixth and seventh generation. In both species the respective cells are situated at the rim of the blastopore just prior to gastrulation: anteriorly in A6.1/A6.1, posteriorly in B7.1/B7.1. While the endodermal fate is already restricted, the descendants of A6.1/A6.1 in both species end up forming the anterolateral part of the intestinal larval tract, whereas cells B7.1/B7.1 show a slightly different migration pattern between the two species (Additional files 1 and 2). In the larvacean the descendants of B7.1/B7.1 form part of the endodermal strand (shown for B7.1 in Additional file 2). whereas in the ascidian embryo the descendants of B7.1/B7.1 form ventral trunk endoderm and it is B7.2 that eventually gives rise to endodermal strand cells (shown for B7.1 and B7.2 in Additional file 2). Similarly, the prospective epidermis cells in the ectodermal lines a6.6/a6.6, a6.8/a6.8, b6.6/b6.6, b5.4/b5.4, the prospective notochordal cells A7.3/A7.3, and the prospective neural tube cells A8.15/A8.15 correspond to each other in the two species in their respective relative positions, their fate restrictions, and their locations in the cell lineage trees (Figs. 1 and 2; Additional files 3, 4, 5, 6, 7, 8, 9 and 10).

### Heterochronic shifts

Besides cases of exact correspondences, however, in some other cell lines correspondences are more relaxed. While, for example, the relative positions in the cell lineage trees of muscle cell lines, pericardium cell lines, and germ cells are similar, fate restrictions occur comparatively later in the ascidian (Figs. 1 and 2; Additional files 11, 12 and 13). For example, the germ cell line is the sister lineage to a line that gives rise to pericardium and anterior somatic muscle cells in both species. This separation, however, occurs at the sixth generation in the larvacean (B6.4/B6.4) and at the seventh generation in the ascidian (B7.6/B7.6) (Fig. 1). If the supposition mentioned above that descendants of B5.1/B5.1 might in fact be entirely endodermal, this would be another case of an earlier fate restriction, interestingly with the daughter cells B6.1/B6.1 still being a case of exact correspondence. This general trend towards a relatively earlier fate restriction in the larvacean embryo leads to a tighter coupling of cell lineage and fate restrictions in *O. dioica*. For example, notochord cells in the left side derive from A6.2 in the larvacean, but from A7.3, A7.7, and B8.6 in the ascidian (Figs. 1 and 2). Similar patterns can be seen in the fate restrictions of nervous system, endoderm, and musculature. These cases where a specific, cohesive larval tissue originates in several separate cell lines indicate in the bifurcating cell lineage tree representation that cell fates might depend on regional cellular interactions in the ascidian, as has been shown in the ascidian *Ciona intestinalis* [27]. While our data are compatible with the hypothesis that in larvaceans cell lineage determines fate to a large extent, regional inductions are not ruled out, but should be tested in laboratory experiments.

### Morphogenetic movements - gastrulation

**Fig. 3:**
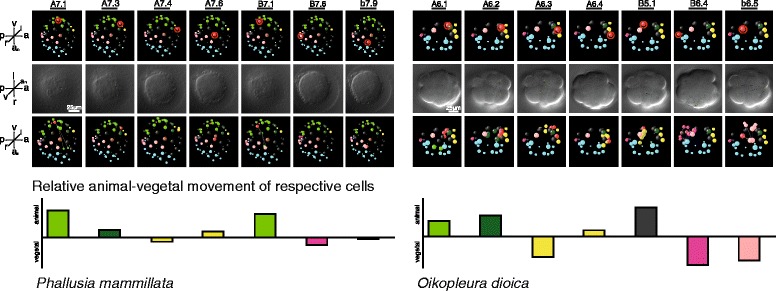
To the *left* the ascidian embryo at 235 min post fertilization (pf) consists of 64 cells. To the *right* the larvacean embryo at 80 min pf consists of 30 cells. The individual cells in each column are named and marked *red* in the respective schematic representation. *Upper row*: schematic representation of embryo at the late blastula stage in lateral view from the right side, anterior is to the right of the image. *Middle row*: differential interference contrast images marking the nucleus of the respective cell, in vegetal view, anterior is to the right of the images. *Lines* mark cell movement to the next generation of cells, i.e., to the next mitosis until the following one. *Lower row*: same images as in the upper row, but with the movement as described for the middle row overlain. This lateral view maximizes the relative visible movements of the cells. *Graph below images*: Movement profile of individual cells at the onset of gastrulation. Relative distances are indicated as measured directly from lower row of images. Measured distances are distances moved along the animal–vegetal axis from start to end of lines shown in middle row. Axis orientation label: *a* anterior, *a*_*n*_ animal, *l* left, *p* posterior, *r* right, *v* vegetal

We also analyzed complex morphogenetic movements such as gastrulation at single cell resolution by comparing cellular movement profiles (Fig. 3). The starting point of these profiles are the precise positional relations of individual cells but because of the lower resolution in time cellular movements within a certain time bracket can be determined only roughly. Nevertheless, we found that the general movement patterns of individual cells that are positioned in a corresponding way around the blastopore at the onset of gastrulation (upper row in Fig. 3) are highly similar, but that the movement occurs one to two rounds of divisions earlier in the larvacean. Moreover, identities according to cell genealogy are not conserved in each case even when position and fate are retained, revealing again evolutionary changes. For example, in the case of cells A6.3 in larvacean and A7.4 in ascidian, we show that the cells are situated at the posterior end of the embryo at the onset of gastrulation (Fig. 3) and give rise to nervous system cells in the neural tube (Additional files 7, 8 and 9) but differ in their location in the cell lineage trees as indicated by their names and in their respective positions in the larval nervous systems.

### Morphogenetic movements - neurulation

**Fig. 4:**
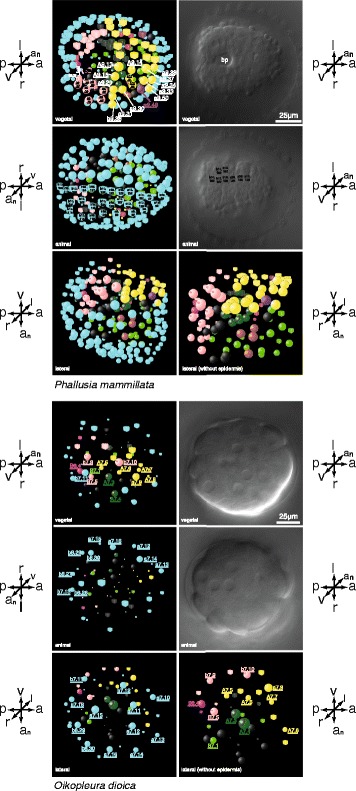
Differential interference contrast images and schematic cell position representations at comparable late gastrula stages in an ascidian embryo (*upper part*) and in a larvacean embryo (l*ower part*). Axis orientation labels next to images indicate orientations: *a* anterior, *a*_*n*_ animal, *l* left, *p* posterior, *r* right, *v* vegetal. Absolute age is indicated by the *blue dotted lines* in Fig. 1; tissue fates are color coded as in Fig. 1. Note the similarity in the relative positions of the different tissues, despite the considerably lower cell number in the larvacean. Note also that prospective nervous system cells (*yellow*) are arranged as a neural plate at the anterior border of the blastopore in the ascidian whereas the corresponding cells have already ingressed (neurulated) in the larvacean. (For detailed spatial and temporal resolution of neural fate restrictions in the two species see Additional files 7, 8, 9 and 10). *bp*, blastopore

The morphogenetic process that results in the typical neural tube of chordates is called neurulation ([28, 29]; see also [30]). With the example of neurulation, the spatiotemporal resolution of comparative cell lineage analyses shows that the relative timings of morphogenetic events are also susceptible to evolutionary changes. Whereas in the ascidian embryos neurulation follows gastrulation, the two processes occur almost simultaneously in larvacean embryology. Thus the highly ordered and stereotypic pattern of prospective nervous system cells in a neural plate at the anterior border of the blastopore in ascidian embryos is not present in the larvacean (Fig. 4). Instead the prospective nervous system cells are already arranged to form an elongated rod-like array along the anteroposterior axis during gastrulation that is pronouncedly narrow at the anterior end at cell generation 7 (Fig. 3 and Additional file 9). While, therefore, a similarly stereotyped pattern of the prospective nervous system cells arranged in a neural plate does not occur in the larvacean embryo of a comparable stage (late gastrula; Fig. 4) and the number of cells is considerably smaller, an overall similarity exists. For example, the prospective neural tube cells are similar in their anterodorsal position and their anteroposterior order. Interestingly, the anterior portion of the prospective nervous system in embryos of *O. dioica* is more bulbous than the posterior one very early on during ontogeny (at the eighth cell division, Additional file 10), thus showing a characteristic that occurs in considerably later stages in the ascidian embryo (at the tenth/eleventh generation of the prospective nervous system cells; see the movie available at [31]). An interesting difference occurring during neurulation in the two species is the bilaterally symmetric arrangement of cells in the ascidian larva, whereas in the larvacean embryo the prospective nervous system cells of both sides intermingle and form a complete dorsal neural tube with a lower number of cells than in the ascidian (Additional file 6: Figure S6, Additional file 7: Figure S7, Additional file 8: Figure S8, Additional file 9: Figure S9 and Additional file 10: Figure S10; [20]).

### Loss of sub-lineages and cellular novelties

The juxtaposition of comparable stages in the two tunicates shows that besides the temporal discrepancy of events, the ascidian embryo has undergone eight or nine mitoses in most lineages when neurulation commences, whereas the larvacean has undergone seven. This means that at this stage there are more than twice the number of cells in the ascidian (c = 181) than in the larvacean (c = 63). Concomitant to the higher number of cells, ascidian embryos possess additional tissue types that are not present in larvaceans, such as the trunk lateral cells (TLCs). TLCs are the descendants of cells A7.6/A7.6 and form a conspicuous cluster of lateral mesenchymatic cells in the trunk of tadpole and larval stages of ascidians (Additional file 14). Because other chordates, such as cephalochordates [32–34], hagfishes [35, 36], or ammocoetes [37, 38], possess mesenchymatic cells during early stages in ontogeny, this outgroup comparison indicates that larvaceans are evolutionarily derived in regard to this trait. TLCs, that is, the descendants of cells A7.6/A7.6, have been suggested to be homologous to vertebrate neural crest [39]. Alternatively, Abitua and colleagues hypothesized that cells a9.49/a9.49 and their descendants correspond to vertebrate neural crest cells [40]. Whereas TLCs are absent in larvaceans, a9.49/a9.49 are present but their descendent cells are anterior epidermis cells and not bordering the dorsal anterior neural tube as is the case in the ascidian embryo (Fig. 4). Thus the precise position of the descendants of a9.49/a9.49 in the ascidian tadpole clearly corresponds to the definition of neural crest cells in the zebrafish anatomy ontology (see [41]), therefore supporting the hypothesis suggested by Abitua and colleagues [40].

### Detailed comparisons of individual cells

**Fig. 5:**
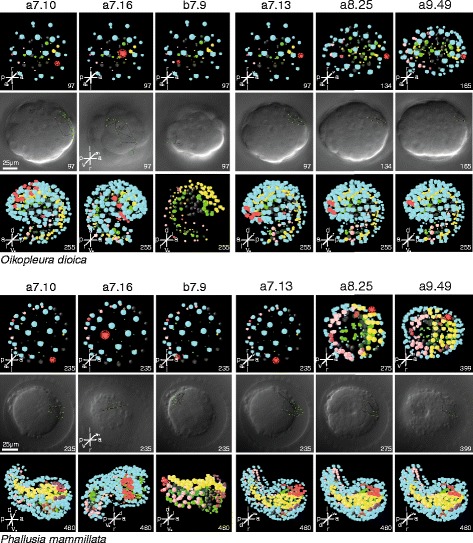
Selected tracings of individual cells from the animal half of the embryo. The cells indicated on top of each column are marked *red* in the schematic representation and the descendants of the respective cells are seen in *red* in the lower rows. The intermediary Differential interference contrast image shows the cell divisions and cell movements of the respective cells. Numbers are minutes post fertilization. Nomarski images are all in the same orientation. Axis orientation labels in images indicate orientations: *a* anterior, *a*_*n*_ animal, *d* dorsal, *l* left, *p* posterior, *r* right, *v* vegetal, *v*_*e*_ ventral. *Upper panel*: *Oikopleura dioica. Lower panel*: *Phallusia mammillata*. The *right-most column* details the mitotic history of cell line a9.49 that has been hypothesized to be homologous to vertebrate neural crest cells

The power of bifocal 4D-microscopy becomes evident when individual cells, their respective fates, and movement patterns at different developmental stages are compared (Fig. 5 and Additional file). For instance, b7.9 and descending cells in the animal half of the gastrulae are situated between prospective epidermis cells yet develop into posterolateral tail muscle cells in both species, although the bending of the tail in relation to the trunk is considerably more pronounced and the tail is affected by an ongoing torsion in the larvacean embryo (Fig. 5, Additional files 3 and 4; see also [20]). In other cases of animal cells, such as a7.10, the descendants of which become anterodorsal epidermis cells in both species, the similarity also comprises the movement pattern from an anterior animal position in the gastrula stage (see Fig. 5). (Numerous and detailed additional examples, also from other germ layers, can be found in the supporting information accompanying this article online). Again, it becomes evident that at all levels certain characteristics are highly similar between the species, whereas others differ to various degrees.

Building on the pioneering study by Conklin on *Styela partita* [25] that has been considerably expanded by Nishida [42, 43] for *Halocynthia roretzi*—who used a tedious injection technique to trace cell fates—several modern studies analyzing cell fates in specific tissues of *Ciona intestinalis* [27, 44–46] documented on the one hand that cell lineages in ascidian species seemed conserved, while stating that “comparative analysis is a difficult exercise due to the current poor anatomical description.” [47]. Our application of bifocal 4D-microscopy for the first time revealed the precise and almost complete cell lineage of an ascidian embryo up to the early tadpole stage (between stage 17 and 18 in the ANISEED database: http://www.aniseed.cnrs.fr/aniseed/anatomy/find_devstage), enabling detailed comparisons with the improved re-analyzed cell lineage of an appendicularian species.

In comparing our detailed documentation of cell lineages of *P. mammillata* with available information on cell lineages in other ascidian species, we found more similarities to the described cell lineages of *C. intestinalis* [27, 44–46] and *H. roretzi* [42, 43] than to the only publication of a cell lineage of *P. mammillata* itself [48]. In their cell fate tabulation (table 1 in [48], p. 196), Zalokar and Sardet combined available data from different species and, using injection of a fluorescent marker in *P. mammillata*, corrected several previous misconceptions [49, 50], notably in the lines becoming muscle cells. For example, these authors realized that cells B7.6/B7.6 do not give rise to muscle cells, but fell short of determining the actual fate of this cell as the germ line cell. This realization came only later in comparative studies using *H. roretzi* as a model [43, 51, 52]. Because other errors had not been corrected and because the study by Zalokar and Sardet remained the only one on the cell lineage of *P. mammillata*, our results are closer to the published cell lineages of *H. roretzi* and *C. intestinalis*. For example, contrary to the lineage published by Zalokar and Sardet [48], A7.6/A7.6 do not give rise to notochord but to TLCs as in *C. intestinalis* [27] and *H. roretzi* [43]. Another example are the B8.6/B8.6 cells, which are tabulated as mesenchymal in fate for *P. mammillata* by Zalokar and Sardet but that we found, in agreement with Nishida [43] and Lemaire [27], become notochordal cells. On the other hand, we note minor differences in some lineages to these studies. Both Nishida and Lemaire describe the fate of B7.7/B7.7 cells as mesenchymal, whereas we found the descendants become muscle and mesenchyme. Whether this discrepancy is due to real differences in the lineages of the different species or due to limitations in resolution remains to be verified. The fine grain of our cell lineage observations also allows us to relate these data with the even finer morphological detail of confocal laser scanning microscopy-derived morphological descriptions of certain ascidian embryonic stages that recently became available [53, 54].

### Evolutionary considerations

Although the exact phylogeny of Tunicata remains controversial [15, 55–57], most paleontological studies indicate that the diversification of Tunicata dates back to the early Cambrian (ca. 525 Mya) [58]. Thus, the precision of our cell lineage data opens an entirely new horizon for evolutionary analyses and interpretation. We could reveal detailed similarities with single cell resolution pertaining to different levels of potential homologies, such as cleavage tree pattern, fate restriction, morphogenetic movements, timing, or orientation of planes of cell divisions. We demonstrated that each of these levels is susceptible to changes and conclude that there is no a priori reason to expect one level to be less prone to evolution than another. Indeed the many levels of evolvability of embryonic development are a stark reminder that evolutionary analyses cannot afford to neglect organismal levels.

### Cell lineage indicates cellular signaling

Detailed cell lineage data can moreover serve as a high-precision guide to devise experimental approaches to investigate other levels as well, such as molecular interactions between cells, changes in gene expression, but also studies designed to unravel the accompanying molecular changes underlying the documented structural evolutionary changes. For example, from the cell lineage tree we would predict that the determination of nervous system cell fate in several of the comparatively late restricting nervous system cell lines such as a8.17/a8.17 and a8.19/a8.19 (descendants of a4.2/a4.2) depend on regional factor signaling whereas this might not be the case for A7.4/A7.4 (descendants of A4.1/A4.1). And indeed, because neural cell lineages in ascidians were available relatively early on [46], it could be demonstrated that neural descendants of A4.1/A4.1 develop autonomously, whereas signaling from this source is necessary for the a4.2/a4.2 descendants to assume a neural fate [47]. We predict that regional induction is similarly necessary for neural fate determination in A8.15/A8.15. Moreover, we argue that the restriction to neural fate in a7.9/a7.9 in the larvacean embryo depends on regional signaling as well and that the reduction of such signaling in several cell lines led to the streamlined cell lineage tree documented in the larvacean *O. dioica*.

## Conclusion

Bifocal 4D-microscopy overcomes the limitations imposed on light microscopic observations in ascidian embryos by the extensive extra embryonic covers of a developing embryo. While many molecular studies remove these coverings [51], removing these covers results in some ontogenetic changes, such as shape of epidermis cells and formation of larval tunic, compared to the wild-type development [59, 60]. Thus bifocal 4D-microscopy can amend these shortcomings and considerably expand our knowledge of tunicate ontogeny at a detailed cellular level. While the present paper focuses on the comparative findings rather than on the methodological advancement, it is obvious that 4D-microscopy in a comparative framework allows for and necessitates new analytical tools and poses new and exciting challenges [26, 61]. An important challenge relates to the notion of homology: with the huge amount of detailed and precise information present in the accumulated data, it becomes obvious that we need to develop software tools that can guide us through the many layers of similarities, including the positional relational information, the movement and cleavage pattern, temporal information of all the observed events, fate restriction patterns, and similarities in underlying gene regulatory networks, and visualize or quantify similarity arguments in support of different homology hypotheses. Because homology is the central concept in evolutionary comparative biology, addressing this challenge with appropriate data mining and analytical tools has to become a priority in the burgeoning field of visual computer analyses.

## Methods

Fundamentals of 4D-microscopy are described by Schnabel et al. [11]. Individual *O. dioica* embryos recorded by Stach et al. [20] were re-analyzed comparatively for the present study. (In contrast to ascidian embryos, *O. dioica* embryos do not possess follicle or test cells and therefore conventional 4D-microscopy suffices.) Four-cell, eight-cell, or sixteen-cell embryos of *P. mammillata* were recorded using bifocal 4D-recordings. Bifocal 4D-microscopy was developed by Dr. Ralf Schnabel (Technical University, Braunschweig) in cooperation with Zeiss. Essentially, in a Zeiss Examiner (Zeiss, Jena, Germany) D1 microscope the condenser was replaced with a second optical microscope unit, including a second objective and camera (Additional file 15). The microscope was equipped with an extended internal focus drive (500 μm; (Physical Instruments, Karlsruhe, Germany)) used to move the stage to record a z series with two PCO pixelfly cameras, documenting each focal plane from above and below. Images were compressed with a wavelet function (Lurawave) LuraTech, Remscheid, Germany. The microscope was controlled with a software programmed by K. Schulz and R. Schnabel. Embryos were recorded at 18 °C in a thermocontrolled room; in addition the upper objective was equipped with a thermoconstant cooling ring. Recordings of three different embryos stemming from three different parental pairs were analyzed using SIMI°BioCell software (SIMI) Simi Reality Motion Systems, Unterschleissheim, Germany. Starting with a four-cell or eight-cell embryo, 140 images were recorded every 60 s from 70 planes 1.7 μm apart with the two cameras. A complete scan consisted of 1,000 scans, resulting in a database comprising 140,000 images. Of the approximately 16 h of development recorded, 8 h were analyzed in detail. For cell nomenclature, we used Conklin’s system for both species [25] (a translation table to the larvacean nomenclature used by Delsman [62] is given in [20]). For the determination of a cell’s name and for the tree representation of the cell lineages we also used Conklin’s set of rules. The daughter cell that is situated closer to the vegetal pole after mitosis received the lower number and, in the tree depiction, is represented by the outer branch [25, 26]. In cases where both cells are at the same height along the animal–vegetal axis, the cell closer to the anterior received the lower number and is represented by the outer branch in the anterior half oft he embryo; in the posterior half oft he embryo it is the cell closer to the posterior that received the lower number and is represented by the outer branch. In cases where the daughter cells are also identical in their respective position along the antero-posterior axis, the more distal cell was given the lower number and represented by the outer branch (see figures 133 and 134 in [25]). To distinguish between the cells from both sides, we again used Conklin’s suggestion and underlined cell names referring to the cells on the right side of the embryo.

*P. mammillata* adults were obtained through the service Modèles Biologiques (ModBio) from the Centre de Resources Biologiques Marines at the Station Biologique Roscoff (France). In each experiment, two individuals were cross-fertilized in vitro as described [51] and normally developing embryos were mounted for microscopic analysis. The latter citation reports results from cell lineage tracing in *P. mammillata*. Our detailed documentation of cell lineages of this very species shows more similarities to the described cell lineages of *C. intestinalis* and *H. roretzi* than to the description of the cell lineage of *P. mammillata* [51]. Complete datasets including complete stacks of differential interference contrast images and SIMI°BioCell-files are deposited on www.morphdbase.de along with Additional files and can be downloaded from there.

### Availability of supporting data

The complete datasets supporting the results of this article are available in the MorphDBase repository as zipped archives.

The database consisting of approximately 140,000 images of a recording of the ontogenetic development of a *Phallusia mammillata* embryo has been separated into two packages, representing the images from the upper and lower camera respectively.

zip-compressed files of the complete scan of the development of *P. mammillata*, including Simi-Biocell analysis files:

www.morphdbase.de/?T_Stach_20151112-M-47.1

www.morphdbase.de/?T_Stach_20151112-M-46.1

zip-compressed file of complete scan of the development of *O. dioica*, including Simi-Biocell analysis files:

www.morphdbase.de/?T_Stach_20151112-M-45.1

A supplementary movie at www.morphdbase.de/?T_Stach_20151020-M-13.1 shows the ontogeny and detailed cell lineages of the two tunicate species *P. mammillata*, an ascidian, and *O. dioica*, a larvacean. The two species have drastically different life histories and ecologies, yet the cell lineage pattern shows remarkable similarities.

Additional movies of tracings of specific cells can be requested from the first author.

High resolution versions of supplementary figures are available as indicated in the captions to the additional files.

## Additional files

Additional file 1:
***Phallusia mammillata*** **.** Analytical cell lineage tracing of individual endodermal cells between blastula stage (3 h 55 min pf) and early tadpole stage (10 h 6 min pf). A higher resolution version of this figure is hosted on MorphDBase at: www.morphdbase.de/?T_Stach_20151119-M-58.1. (PDF 4316 kb) Additional file 2:
***Oikopleura dioica*** **.** Analytical cell lineage tracing of individual endodermal cells between blastula stage (1 h 37 min pf) and early larval stage (4 h 15 min pf). A higher resolution version of this Fig. is hosted on MorphDBase at: www.morphdbase.de/?T_Stach_20151119-M-59.1. (PDF 2348 kb) Additional file 3:
***Phallusia mammillata*** **.** Analytical cell lineage tracing of individual animal-half cells between blastula stage (3 h 55 min pf) and early tadpole stage (10 h 6 min pf). A higher resolution version of this figure is hosted on MorphDBase at: www.morphdbase.de/?T_Stach_20151119-M-60.1. (PDF 5844 kb) Additional file 4:
***Oikopleura dioica*** **.** Analytical cell lineage tracing of individual animal-half cells between blastula stage (1 h 37 min pf) and early larval stage (4 h 15 min pf). A higher resolution version of this figure is hosted on MorphDBase at: www.morphdbase.de/?T_Stach_20151119-M-61.1. (PDF 5425 kb) Additional file 5:
***Phallusia mammillata*** **.** Analytical cell lineage tracing of individual notochordal cells between blastula stage (3 h 55 min pf) and early tadpole stage (10 h 6 min pf). A higher resolution version of this figure is hosted on MorphDBase at: www.morphdbase.de/?T_Stach_20151119-M-62.1. (PDF 6767 kb) Additional file 6:
***Oikopleura dioica*** **.** Analytical cell lineage tracing of individual notochordal cells between blastula stage (1 h 17 min pf) and early larval stage (4 h 15 min pf). A higher resolution version of this figure is hosted on MorphDBase at: www.morphdbase.de/?T_Stach_20151119-M-63.1. (PDF 2830 kb) Additional file 7:
***Phallusia mammillata*** **.** Analytical cell lineage tracing of individual nervous system cells between blastula stage (3 h 55 min pf) and early tadpole stage (10 h 6 min pf). A higher resolution version of this figure is hosted on MorphDBase at: www.morphdbase.de/?T_Stach_20151119-M-64.1. (PDF 2998 kb) Additional file 8:
***Phallusia mammillata***. Analytical cell lineage tracing of individual nervous system cells between gastrula stage (5 h 38 min pf) and early tadpole stage (10 h 6 min pf). A higher resolution version of this figure is hosted on MorphDBase at: www.morphdbase.de/?T_Stach_20151119-M-65.1. (PDF 8904 kb) Additional file 9:
***Oikopleura dioica*** **.** Analytical cell lineage tracing of individual nervous system cells between earlier blastula stage (57 min pf)and early larval stage (4 h 15 min pf). A higher resolution version of this figure is hosted on MorphDBase at: www.morphdbase.de/?T_Stach_20151119-M-66.1. (PDF 4700 kb) Additional file 10:
***Oikopleura dioica*** **.** Analytical cell lineage tracing of individual nervous system cells between later gastrula stage (2 h 12 min pf) and early larval stage (4 h 15 min pf). A higher resolution version of this figure is hosted on MorphDBase at: www.morphdbase.de/?T_Stach_20151119-M-67.1. (PDF 3697 kb) Additional file 11:
***Phallusia mammillata*** **.** Analytical cell lineage tracing of individual muscle cells between blastula stage (3 h 55 min pf) and early tadpole stage (10 h 6 min pf). A higher resolution version of this figure is hosted on MorphDBase at: www.morphdbase.de/?T_Stach_20151119-M-68.1. (PDF 4703 kb) Additional file 12:
***Phallusia mammillata*** **.** Analytical cell lineage tracing of individual muscle cells between gastrula stage (5 h 38 min pf) and early tadpole stage (10 h 6 min pf). A higher resolution version of this figure is hosted on MorphDBase at: www.morphdbase.de/?T_Stach_20151119-M-69.1. (PDF 5654 kb) Additional file 13:
***Oikopleura dioica*** **.** Analytical cell lineage tracing of individual muscle cells between blastula stage (1 h 17 min pf) and early larval stage (4 h 15 min pf). A higher resolution version of this figure is hosted on MorphDBase at: www.morphdbase.de/?T_Stach_20151119-M-70.1. (PDF 3696 kb) Additional file 14:
***Phallusia mammillata*** **.** Analytical cell lineage tracing of individual trunk lateral cells (TLCs) between blastula stage (3 h 55 min pf) and early tadpole stage (10 h 6 min pf). A higher resolution version of this figure is hosted on MorphDBase at: www.morphdbase.de/?T_Stach_20151119-M-71.1. (PDF 721 kb) Additional file 15:
**A Bifocal 4D-microscope.** B Higher magnification of the lower objective that replaces the condenser in the bifocal 4D-microscope. C Adult *Phallusia mammillata*. D–G Pictures of removal of gametes from a hermaphroditic *P. mammillata* individual during the fertilization process in the laboratory. A higher resolution version of this figure is hosted on MorphDBase at: www.morphdbase.de/?T_Stach_20151119-M-72.1. (PDF 3653 kb)
