# Supplementary material for: High-precision morphology: bifocal 4D-microscopy enables the comparison of detailed cell lineages of two chordate species separated for more than 525 million years
Source: BMC Biol. 2015 Dec 23;13:113. doi: 10.1186/s12915-015-0218-1 (PMC4690324; doi:10.1186/s12915-015-0218-1)
Supplement: Additional file 3: — Phallusia mammillata . Analytical cell lineage tracing of individual animal-half cells between blastula stage (3 h 55 min pf) and early tadpole stage (10 h 6 min pf). A higher resolution version of this figure is hosted on MorphDBase at: www.morphdbase.de/?T_Stach_20151119-M-60.1. (PDF 5844 kb) [file 12915_2015_218_MOESM3_ESM.pdf]

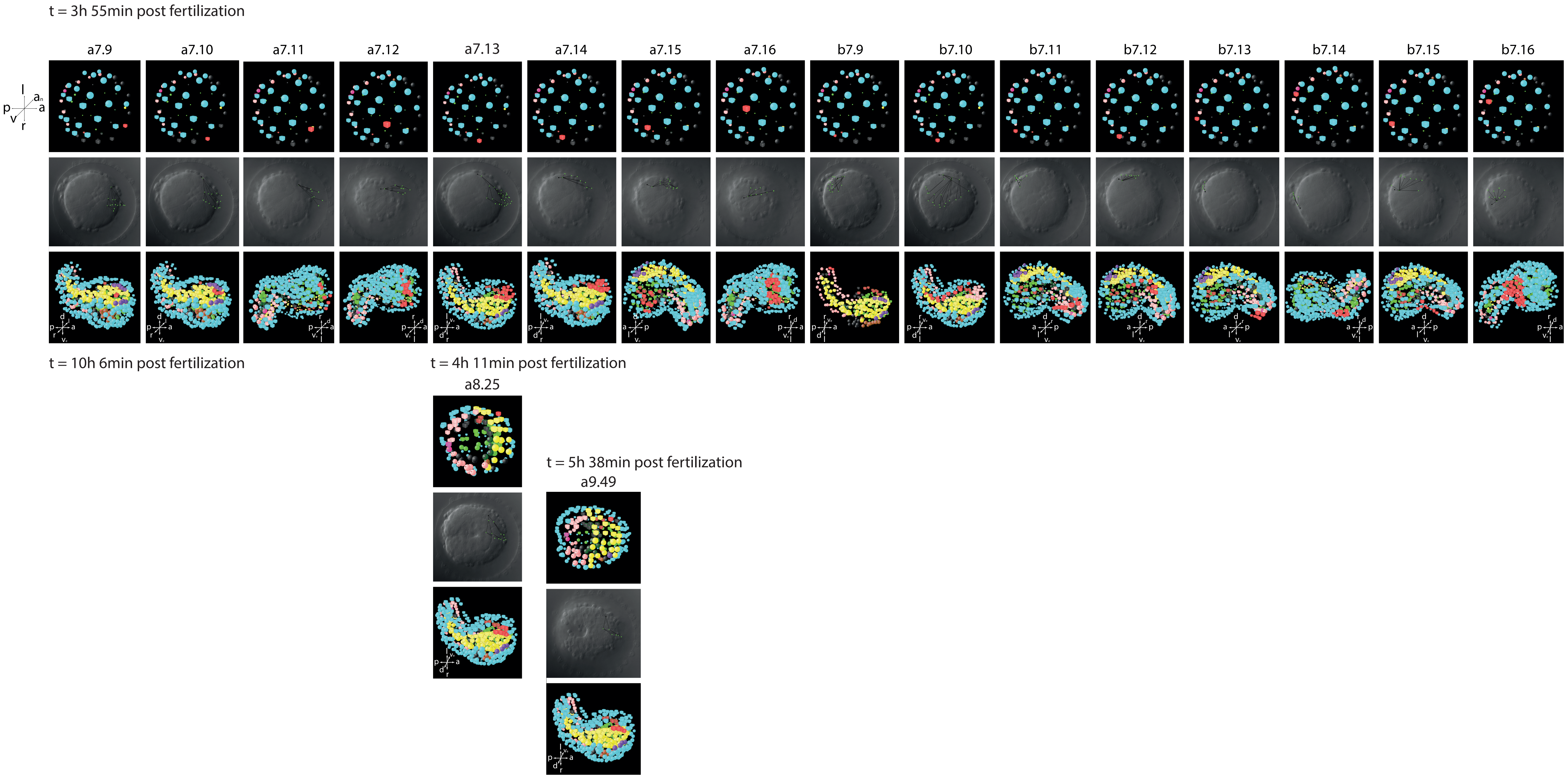

Supplementary Figure 3. *Phallusia mammillata*. Single cell analysis of cells from the animal side of the embryo at different times of development. Individual cells are marked red in the blastula stages (t = 3h, 55 min after fertilization at 18°C) in the schematic 3D-representations in the top rows. Rows with Nomarski images show changes in cell position in consecutive generations documented in the 4D-analysis. Schematic 3D-representation in lower row shows descendants of individual cell in the respective column. a7.13-line is the potential neural crest cell line as hypothesized by Abitua et al. (2012). (some descendants might be missing). In all images the trunk of the embryo is oriented as depicted in the axis-orientation labels in the upper left of the figure, unless specified in the respective image. **a** – anterior, **an** – animal, **d** – dorsal, **l** – left, **p** – posterior, **r** – right, **v** – vegetal, **ve** – ventral.
A higher resolution version of this figure is hosted on MorphDBase at: www.morphdbase.de/?T_Stach_20151119-M-60.1
