# Supplementary material for: High-precision morphology: bifocal 4D-microscopy enables the comparison of detailed cell lineages of two chordate species separated for more than 525 million years
Source: BMC Biol. 2015 Dec 23;13:113. doi: 10.1186/s12915-015-0218-1 (PMC4690324; doi:10.1186/s12915-015-0218-1)
Supplement: Additional file 6: — Oikopleura dioica . Analytical cell lineage tracing of individual notochordal cells between blastula stage (1 h 17 min pf) and early larval stage (4 h 15 min pf). A higher resolution version of this figure is hosted on MorphDBase at: www.morphdbase.de/?T_Stach_20151119-M-63.1. (PDF 2830 kb) [file 12915_2015_218_MOESM6_ESM.pdf]

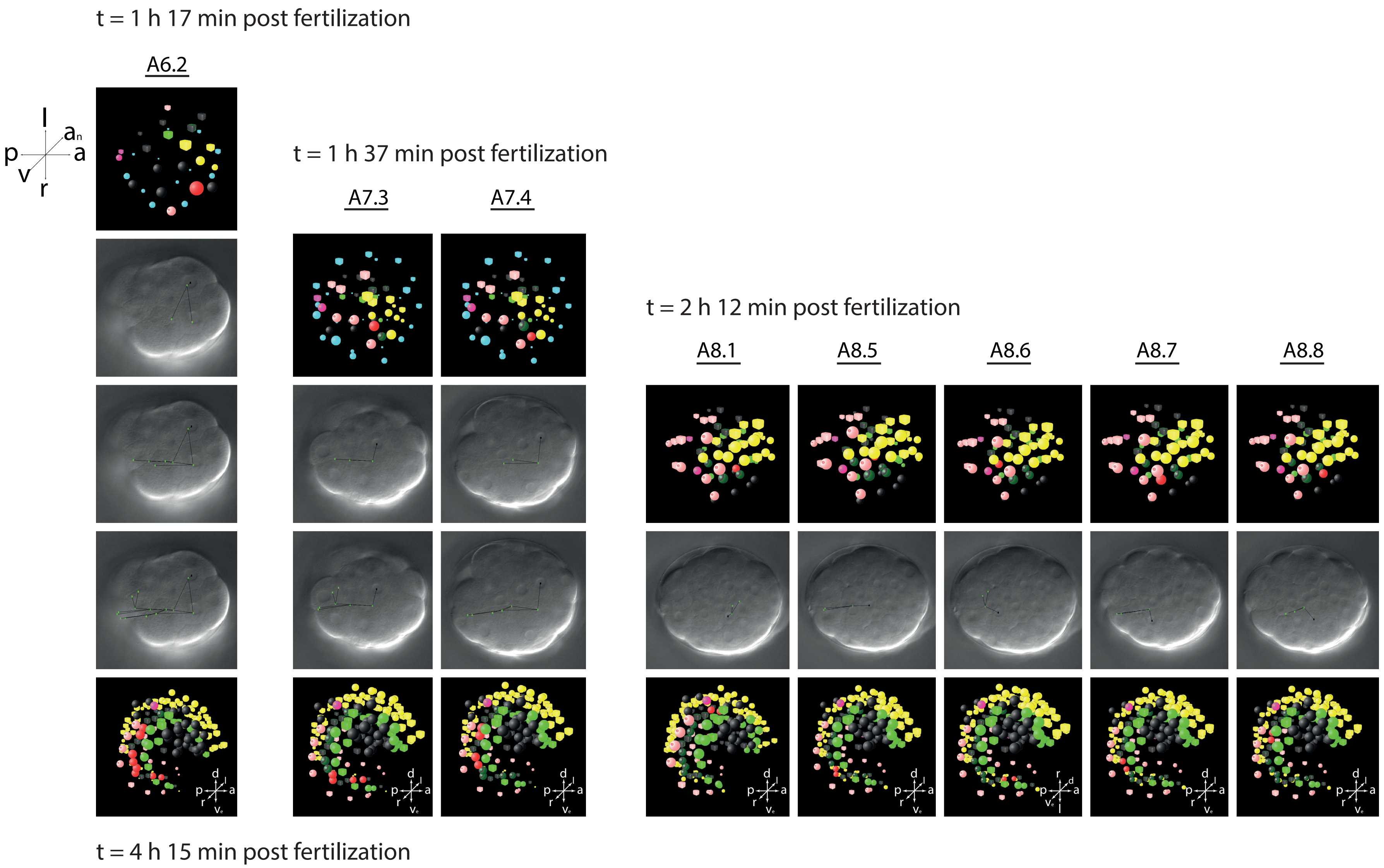


**Supplementary Figure 6**. *Oikopleura dioica*. Single cell analysis of notochord cells at different times of development. Individual cells are marked red in the schematic 3D-representations in the top rows. Rows with Nomarski images show changes in cell position in different numbers of consecutive generations, starting with 1 in the first image of the respective column. Schematic 3D-representation in lower row shows decendants of individual cell in the respective column (some descendants might be missing). In all images the trunk of the embryo is oriented as depicted in the axis-orientation labels in the upper left of the figure, unless specified in the respective image.

**a** – anterior, **an** – animal, **d** – dorsal, **l** – left, **p** – posterior, **r** – right, **v** – vegetal, **ve** – ventral.

A higher resolution version of this figure is hosted on MorphDBase at: www.morphdbase.de/?T_Stach_20151119-M-63.1
