# Supplementary material for: High-precision morphology: bifocal 4D-microscopy enables the comparison of detailed cell lineages of two chordate species separated for more than 525 million years
Source: BMC Biol. 2015 Dec 23;13:113. doi: 10.1186/s12915-015-0218-1 (PMC4690324; doi:10.1186/s12915-015-0218-1)
Supplement: Additional file 12: — Phallusia mammillata . Analytical cell lineage tracing of individual muscle cells between gastrula stage (5 h 38 min pf) and early tadpole stage (10 h 6 min pf). A higher resolution version of this figure is hosted on MorphDBase at: www.morphdbase.de/?T_Stach_20151119-M-69.1. (PDF 5654 kb) [file 12915_2015_218_MOESM12_ESM.pdf]

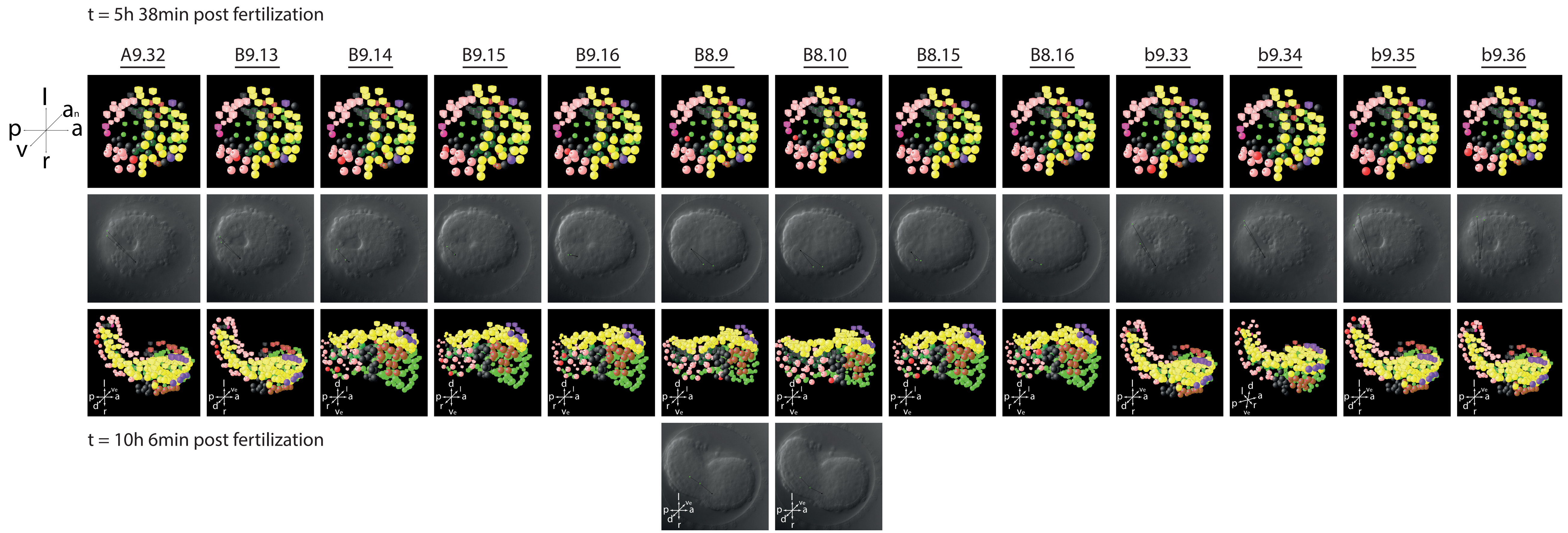

Supplementary Figure 12. *Phallusia mammillata*. Single cell analysis of muscle cells at different times of development. Individual cells are marked red in the schematic 3D-representations in the top rows. Rows with Nomarski images show changes in cell position in different numbers of consecutive generations, starting with 1 in the first image of respective column. Schematic 3D-representation in lower row shows decendants of individual cell in the respective column (some descendants might be missing). B8.9 and B8.10 give rise to anterior muscle cells and in addition probably to pericardium ("heart") cells. The additional Nomarski images in the lowest row show the movements of the previous (!) generation of the probable pericardium cells. In all images the trunk of the embryo is oriented as depicted in the axis-orientation labels in the upper left of the figure, unless specified in the respective image. **a** – anterior, **an** – animal, **d** – dorsal, **l** – left, **p** – posterior, **r** – right, **v** – vegetal, **ve** – ventral. A higher resolution version of this figure is hosted on MorphDBase at: www.morphdbase.de/?T_Stach_20151119-M-69.1
