# Supplementary material for: High-precision morphology: bifocal 4D-microscopy enables the comparison of detailed cell lineages of two chordate species separated for more than 525 million years
Source: BMC Biol. 2015 Dec 23;13:113. doi: 10.1186/s12915-015-0218-1 (PMC4690324; doi:10.1186/s12915-015-0218-1)
Supplement: Additional file 14: — Phallusia mammillata . Analytical cell lineage tracing of individual trunk lateral cells (TLCs) between blastula stage (3 h 55 min pf) and early tadpole stage (10 h 6 min pf). A higher resolution version of this figure is hosted on MorphDBase at: www.morphdbase.de/?T_Stach_20151119-M-71.1. (PDF 721 kb) [file 12915_2015_218_MOESM14_ESM.pdf]

t = 3h 55min 10s post fertilization

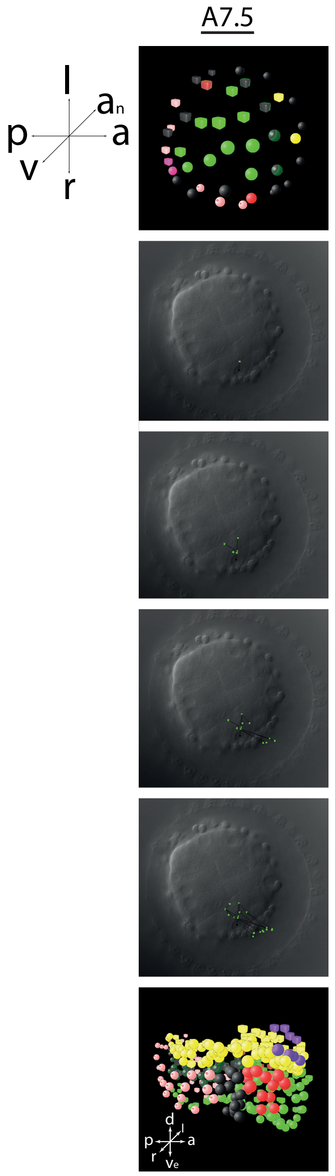

t = 10h 6min 40s post fertilization

Supplementary Figure 14. *Phallusia mammillata*. Single cell analysis of trunk lateral cell lineage. A7.5 is marked red in the schematic 3D-representation in the top row. Rows with Nomarski images show changes in cell position in different numbers of consecutive generations, starting with 1 in the first image of the column. Lower schematic 3D-representation shows descendants of A7.5 at 10h, 6min after fertilization at 18 °C. In all images the trunk of the embryo is oriented as depicted in the axis-orientation labels in the upper left of the figure, unless specified in the respective image. **a** – anterior, **an** – animal, **d** – dorsal, **l** – left, **p** – posterior, **r** – right, **v** – vegetal, **ve** – ventral.
A higher resolution version of this figure is hosted on MorphDBase at: www.morphdbase.de/?T_Stach_20151119-M-71.1
