# Supplementary material for: High-precision morphology: bifocal 4D-microscopy enables the comparison of detailed cell lineages of two chordate species separated for more than 525 million years
Source: BMC Biol. 2015 Dec 23;13:113. doi: 10.1186/s12915-015-0218-1 (PMC4690324; doi:10.1186/s12915-015-0218-1)
Supplement: Additional file 15: — A Bifocal 4D-microscope. B Higher magnification of the lower objective that replaces the condenser in the bifocal 4D-microscope. C Adult Phallusia mammillata. D–G Pictures of removal of gametes from a hermaphroditic P. mammillata individual during the fertilization process in the laboratory. A higher resolution version of this figure is hosted on MorphDBase at: www.morphdbase.de/?T_Stach_20151119-M-72.1. (PDF 3653 kb) [file 12915_2015_218_MOESM15_ESM.pdf]

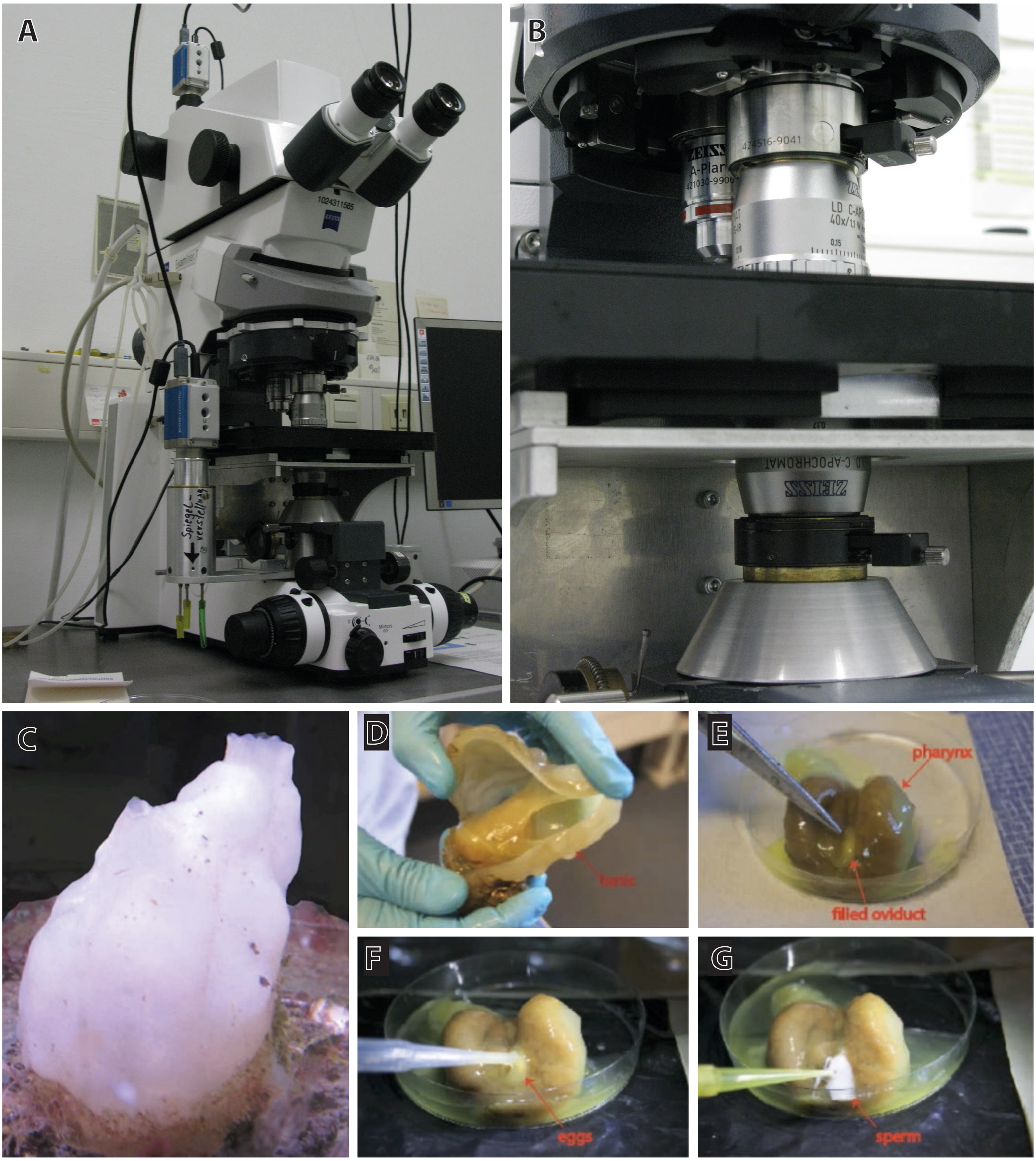

Supplementary Figure 15. **A** – Bifocal 4D-microscope. Note the two cameras on top of the microscope and to the left. Note also that the condensor is replaced by a second objective.
B – Higher magnification of the lower objective that replaces the condensor in the bifocal 4D-microscope. Starting with a 4-cell embryo or an 8 cell embryo, every 60 seconds 140 images from 70 planes 1.7 µm apart were recorded with the two cameras. A complete scan consisted of 1000 scans, resulting in a database comprising 140000 images. Of the resulting approximately 16 hours of development recorded, 8 hours were analyzed in detail. **C** – Adult *Phallusia mammillata*.
D-G – Pictures of removal of gametes from a hermaphroditic *P. mammillata* individual during the fertilization process in the laboratory.
A higher resolution version of this figure is hosted on MorphDBase at:
www.morphdbase.de/?T_Stach_20151119-M-72.1
